# Infectious disease complications associated with opioid use disorder at a southern county hospital: a retrospective chart review

**DOI:** 10.1186/s12879-025-12387-z

**Published:** 2026-01-17

**Authors:** Harini Balakrishnan, Nicholas Campalans, Kapila Marambage, Joan Reisch, Ank E. Nijhawan

**Affiliations:** 1 University of Texas Southwestern Medical School, Dallas, TX USA; 2 Department of Internal Medicine, University of California San Francisco Medical Center, San Francisco, CA USA; 3 Department of Psychiatry, University of Texas Southwestern Medical Center, Dallas, TX USA; 4 O’Donnell School of Public Health, University of Texas Southwestern Medical Center, 5323 Harry Hines Blvd, Dallas, TX USA; 5 Department of Internal Medicine, Division of Infectious Diseases and Geographic Medicine, University of Texas Southwestern Medical Center, Dallas, TX USA

**Keywords:** Opioid use, Injection drug use, Infections, Hospitalization, Medication for opioid use disorder

## Abstract

**Background:**

Injection drug-use (IDU) is related to significant morbidity and mortality. Recent studies of IDU-associated infections have primarily focused on regions outside of the southern US. However, this area presents greater barriers to healthcare funding and lower availability of harm reduction services such as syringe service programs. In this study, we sought to describe infectious disease burden, uptake of medication for opioid use disorder (MOUD), and healthcare utilization in patients with opioid use disorder (OUD) who received inpatient addiction psychiatry consultation at a large safety-net hospital in the southern US.

**Methods:**

A retrospective electronic health record review was conducted for patients admitted to an urban county hospital from February 2018-February 2020. Inclusion criteria: (a) OUD within the last 12 months and (b) addiction psychiatry consultation. Baseline characteristics were compared between those with and without IDU-associated infections. IDU-associated infections, cultured microbes, and MOUD uptake were described.

**Results:**

Of 283 charts reviewed, 248 individuals met inclusion criteria. Overall, 65% were male, 48% non-Hispanic white, and 34% Hispanic. In total, 72% reported opioid IDU in the past 30 days and 52% had IDU-associated infections, including skin and soft-tissue infections (SSTI) (49%), bacteremia (17%), osteomyelitis, (7%), and endocarditis (5%). Comorbid stimulant use disorder was frequently reported (70%). Methicillin-resistant *Staphylococcus aureus* was the most common organism identified (24%). Though 40% of IDU-associated infections were monomicrobial, 23% were polymicrobial (including anaerobes, gram-negatives, and yeast species). Those with IDU-associated infections had lower rates of MOUD on admission (8% vs. 32%) but had higher new MOUD uptake (81% vs. 51%).

**Conclusions:**

SSTIs, which were often polymicrobial, comprised most IDU-associated infections among inpatients with OUD hospitalized in an urban Texas hospital. Individuals with IDU-associated infections had increased MOUD uptake. These findings have implications for empiric antibiotic management of IDU-associated infections and support inpatient initiation of substance use treatment.

## Background

The opioid epidemic affects millions of Americans annually. In 2019, an estimated 10.1 million people aged 12 or older had misused opioids in the past year, of whom 745,000 used heroin [1]. Overdose deaths in the US have continued to escalate through 2022, with increases in fentanyl and concomitant stimulant use [2]. Furthermore, rates of infections in people who inject drugs (PWID) have increased over the past two decades [3–5]. Increases in infections such as endocarditis, HIV, Hepatitis C Virus (HCV), skin and soft-tissue infections (SSTIs), and osteomyelitis result in a significant degree of morbidity among PWID and are associated with high rates of hospitalization, recurrent infection, and death [6, 7]. Upward trends in hospitalization for PWID are accompanied by an almost quadruple increase in charges, as PWID may require long treatment courses and are typically restricted from outpatient parenteral antimicrobial therapy (OPAT) programs [8]. In addition, inpatients with injection drug use (IDU)-associated infections often receive sub-optimal treatment for opioid use disorder (OUD), which may contribute to patient-directed discharge, incomplete antibiotic treatment courses, and readmissions [7].

Hospitalization is a critical time to provide comprehensive medical care to PWID: this group may limit their engagement with outpatient or preventive services due to discrimination and stigma [9, 10], and high rates of uninsurance may discourage engaging with the healthcare system in non-life-threatening circumstances [11]. Thus, hospitalization may serve as a “reachable moment” to initiate addiction treatment, such as medication for OUD (MOUD) [12]. For example, a multidisciplinary model in Oregon incorporating an addiction medicine provider, social worker, and peer mentor saw significant rates of MOUD uptake (60%) [12]. The opioid epidemic is a significant driver of infectious disease complications; teams linking both infectious disease and OUD interventions are being developed to mitigate these related public health crises with demonstrated improvement in outcomes. For example, addiction medicine consultation for patients hospitalized with IDU-associated infections has been associated with increased treatment of OUD and reduced readmission rates [13, 14].

Furthermore, while the southern US is not well-represented in studies characterizing infectious disease complications in PWID, IDU-associated infections in patients with OUD can vary regionally as a consequence of drug markets, which may impact drug characteristics such as purity or the presence of adulterants [15]. Fentanyl demonstrates a significant and growing problem in Dallas and Texas more broadly; however, black tar heroin, which cannot be easily mixed with fentanyl, is most common in Texas [16–18]. Cities with greater historic availability of black tar heroin, such as Seattle or San Francisco, experience double the rate of skin and soft tissue infections compared to those with white-powder heroin [15]. Black tar heroin’s stickiness requires repeated flushing of syringes, possibly accounting for lower transmission rates of HIV, but higher rates of SSTIs secondary to nonsterile water use [19]. Furthermore, its impure nature can cause venous sclerosis via intravenous injection, leading to intramuscular and subcutaneous injection (i.e. “skin popping”), which facilitates anaerobic bacterial growth [20].

In addition, regional differences in healthcare systems present differential barriers to access: PWID are less likely to be insured in Medicaid non-expansion states like Texas (36%) compared to Medicaid expansion states (87%) [21]. Also, despite evidence for harm reduction strategies in the mitigation of IDU-associated infection [12, 22, 23], restrictions, underfunding, inequitable distribution of services, and the prevalence of “abstinence-only” philosophy limits access to syringe service programs [24, 25], which may further impact geographic differences in infections among PWID.

In this study, we aim to describe infectious disease burden, MOUD uptake, and healthcare utilization in patients with OUD who received addiction psychiatry consultation at a southern county hospital. Specifically, we will (1) compare baseline characteristics for patients with OUD who present with IDU-associated infections and those who do not; (2) characterize infectious disease complications related to opioid IDU and associated microbes; (3) compare uptake of MOUD during hospitalization between patients with and without IDU-associated infections; and (4) compare emergency room utilization and hospital readmission at 90 days after discharge between patients with and without IDU-associated infections.

## Methods

This study is a retrospective electronic health record (EHR) review of individuals admitted to Parkland Hospital, an 882-bed urban safety-net hospital, from February 1, 2018 to February 29, 2020. Parkland Health provides care to Dallas County residents, and about half of Parkland’s patients are uninsured [26]. In 2017, a full-time addiction psychiatrist was hired to provide inpatient consultation for hospitalized patients with substance use disorder. Patients also have access to peer navigators who are in recovery from substance use and help facilitate inpatient recovery support groups, as well as a clinical pharmacist and social worker. Informed consent was not obtained from participants as this was determined not to be necessary for this retrospective electronic medical record review which involved only minimal risk to human subjects. This study qualified for exemption from human subjects’ research regulations by the University of Texas Southwestern Medical Center Institutional Review Board. This study adhered to the Declaration of Helsinki regarding research carried out on humans and/or human data.

Eligible patients include those who were: (1) > = 18 years old, (2) received addiction psychiatry consult services, (3) determined to have substance use disorder (documentation of “yes” to the addiction medicine consult order question “Does the patient have a substance use disorder or acute withdrawal that significantly contributes to their current presentation?”), and (4) injecting or non-injecting OUD, defined as an ICD-10 code of F11.x. Patient lists maintained by the addiction consult service were supplemented with patient lists pulled from the hospital EHR system meeting these criteria. Individuals were excluded from analyses if: (1) the patient had no history of OUD or the patient’s OUD was in “sustained remission” for 12 months, as per DSM-5 diagnostic criteria; (2) the patient died during the hospitalization, as outcomes of interest could not be evaluated; or (3) the patient’s record was duplicated. Index admissions were reviewed to confirm active OUD, defined as OUD within the last 12 months.

Data were collected on patient demographics, including age, gender (male or female), race (Black, White, American Indian, or Asian), ethnicity (Hispanic yes/no), housing stability (yes/no), and health insurance status and type (private, Medicaid, Medicare, and self-pay or charity care).

Comorbidities were determined as documentation of the following medical and psychiatric conditions, including diabetes, malignancy, chronic obstructive pulmonary disease, hypertension, congestive heart failure, hepatitis A, hepatitis B, active hepatitis C (defined as a positive RNA test and documentation of active untreated infection by the primary team), human immunodeficiency virus (HIV) status, depression, anxiety, bipolar disorder, psychotic disorder, post-traumatic stress disorder (PTSD), and self-report of trauma. Substance use of cannabis, sedatives/hypnotics/anxiolytics, stimulants, tobacco, and inhalants was determined from the addiction consult team’s notes. Alcohol use disorder was defined by addiction consult team note diagnosis or recorded alcohol consumption of 14 drinks per week for men and 7 for women. Recent opioid IDU, defined as known intramuscular, intravenous, or subcutaneous injection within the last 30 days as documented by provider progress notes in the EHR and using key word searches “IDU, IVDU, injection, intravenous”, as well as any history of IDU were recorded.

Data from hospitalizations were collected, including discharge diagnosis, length of stay, prevalence and type of infection, inpatient administration of intravenous antibiotics, inpatient consult of infectious diseases, and discharge disposition. Infections were divided into acute infections associated with IDU (skin and soft tissue infection, pneumonia, osteomyelitis, endocarditis, bacteremia, epidural abscess, necrotizing fasciitis/myositis, fungemia) and other types of infections (e.g., urinary tract infections, syphilis, oral thrush). Acute infections attributed in the patient’s EMR to a cause unrelated to IDU, such as bacteremia in the setting of colitis or hospital-acquired pneumonia, were classified as other infections. The microorganisms identified in cultures were recorded unless documented as a contaminant.

Patient treatment for OUD was collected. This included MOUD treatment status on admission (defined as receiving MOUD prior to admission), inpatient MOUD uptake (yes/no), and new MOUD uptake (defined as both not receiving MOUD at presentation and newly starting MOUD during this admission), and outpatient OUD treatment referral (referral to a specialty clinic for maintenance treatment). The type of MOUD (methadone, buprenorphine, buprenorphine/naloxone, and naltrexone) was specified. Data on healthcare utilization were collected, including follow-up clinic attendance within the hospital system, frequency of ED visits within 90 days, and rehospitalization (yes/no) within 90 days.

Analytic Plan.

A grouping variable for patients with IDU-associated infections was generated, defined as both (1) recent IDU and (2) presence of one or more IDU-associated acute infections. The comparison group consisted of patients with OUD who (1) did not have recent opioid IDU and (2) were hospitalized for a reason other than an IDU-associated infection. Frequencies (for binary and categorical variables) and means with standard deviations (continuous variables) were used to characterize baseline characteristics and hospital courses of individuals. Comparisons between patients with and without IDU-associated infections were performed using Chi-square contingency table analysis for categorical data or Fisher’s exact test when sample sizes were small and Student’s t test for numerical outcomes. Multivariate logistic regression including demographic and clinical variables was performed to assess (1) the association between IDU-associated infections and MOUD uptake, and (2) the association between IDU-associated infections and rehospitalization within 90 days. For Model 1, the IDU-associated infection grouping variable was included a priori. For Model 2, the IDU-associated infection grouping variable and a binary variable of any MOUD uptake were included a priori. Stepwise selection was used to define final model covariates with an SLentry criteria of 0.05. No adjustments were made for multiple comparisons.

Statistical analysis was performed in SAS, version 9.4 (SAS Institute Inc., Cary, NC).

## Results

Overall, 283 patient charts were reviewed and 248 patients met inclusion criteria. Charts were excluded as follows: 3 patients died during the inpatient hospitalization; 22 patients did not have active OUD; 10 patients were excluded due to duplicated records.

Of the 248 patients included, 64.5% were male, 34.3% were Hispanic, 48.0% were non-Hispanic White, and 17.3% were non-Hispanic Black (Table 1). The majority, 69.8%, were uninsured; while 17.7% of patients were insured by Medicaid. Patients reported high rates of concurrent tobacco use (77.4%) followed by stimulant use (70.2%). Overall, 179 (72.2%) patients reported opioid IDU in the past 30 days and 130 (52.4%) patients were determined to have IDU-associated infections. Patients with IDU-associated infections reported lower rates of stable housing status (35.4% vs. 55.9%, *p* < 0.01) and higher rates of uninsurance (80.0% vs. 58.5%, *p* < 0.01).

**Table 1:** Patients with addiction psychiatry consulted for OUD

Characteristic	All Patients (*N* = 248)	IDU-Associated Infection (*N* = 130)	No IDU-Associated Infection (*N* = 118)	*p* Value
**Age – yr**	40.0 ± 13.1	38.7 ± 12.1	41.4 ± 13.9	*0.105*
**Gender (Male)** ***– no. (%)***	160 (64.5)	86 (66.2)	74 (62.7)	*0.572*
**Race/Ethnicity** ***– no. (%)***				
Non-Hispanic White	119 (48.0)	60 (46.2)	59 (50.0)	*0.545*
Non-Hispanic Black	43 (17.3)	23 (17.7)	20 (16.9)	*0.877*
Hispanic	85 (34.3)	46 (35.3)	39 (33.1)	*0.699*
Other	1 (0.4)	1 (0.8)	0 (0)	*1.000*
**Housing (Stable)** ***– no. (%)***	112 (45.2)	46 (35.4)	66 (55.9)	*0.001*
**Length of Stay – days (mean**,** standard deviation)**	10.5 ± 12.9	10.6 ± 12.6	10.4 ± 13.2	*0.902*
**Insurance Status** ***– no. (%)***				*0.003*
Private	10 (4.0)	3 (2.3)	7 (5.9)	
Medicaid	44 (17.7)	14 (10.8)	30 (25.4)	
Medicare	19 (7.7)	9 (6.9)	10 (8.5)	
Self-Pay/Charity	173 (69.8)	104 (80.0)	69 (58.5)	
**Medical Comorbidities** ***– no. (%)***				
Diabetes	27 (10.9)	11 (8.5)	16 (13.6)	*0.222*
Malignancy	13 (5.2)	3 (2.3)	10 (8.5)	*0.043*
COPD	21 (8.5)	7 (5.4)	14 (11.9)	*0.070*
HTN	69 (27.8)	35 (26.9)	34 (28.8)	*0.677*
CHF	13 (5.2)	7 (5.4)	6 (5.1)	*1.000*
Hep A	2 (0.8)	1 (0.8)	1 (0.8)	*1.000*
Hep B	5 (2.0)	4 (3.1)	1 (0.8)	*0.374*
Hep C	82 (33.1)	45 (34.6)	37 (31.4)	*0.652*
HIV	24 (9.7)	14 (10.8)	10 (8.5)	*0.669*
**Substance Use** ***– no. (%)***				
Cannabis	73 (29.4)	35 (26.9)	38 (32.2)	*0.362*
Sedatives/Hypnotics/Analgesics	72 (29.0)	35 (26.9)	37 (31.4)	*0.442*
Stimulants	174 (70.2)	99 (76.2)	75 (63.6)	*0.030*
Tobacco	192 (77.4)	106 (81.5)	86 (72.9)	*0.103*
Inhalants	7 (2.8)	2 (1.5)	5 (4.2)	*0.246*
Alcohol	67 (27.0)	31 (23.8)	36 (30.5)	*0.238*

Abbreviations: CHF, congestive heart failure; COPD, chronic obstructive pulmonary disease; Hep, hepatitis; HIV, human immunodeficiency virus; HTN, hypertension, IDU, injection drug use; OUD, opioid use disorder

Overall, 130 (52.4%) patients had at least 1 infection associated with IDU (Table 2). SSTIs were most prevalent (48.8%), followed by bacteremia (16.5%), pneumonia (8.5%), osteomyelitis (7.3%), endocarditis (5.2%), fungemia (1.6%), necrotizing fasciitis/myositis (1.2%), and epidural abscesses (0.4%), and all of these were more common among patients with recent IDU. Of patients with IDU-associated infections, 40.0% had monomicrobial infections, 23.1% had polymicrobial infections, and 36.9% did not have an organism identified on culture (Table 3). Of the identified microbes, methicillin-resistant *Staphylococcus aureus* was found at the highest frequency (23.8%), but anaerobes, gram-negatives, and yeast species were also identified. In addition, 5.4% of patients grew *Clostridium spp.*, 4.6% grew *Klebsiella pneumoniae*, and 5.4% grew *Candida spp*.

**Table 2:** Acute and chronic infections in patients with OUD

	All Patients (*N* = 248)	IDU-Associated Infection (*N* = 130)	No IDU-Associated Infection (*N* = 118)
**Acute Infection – no. (%)**			
SSTI	121 (48.8)	106 (81.5)	13 (11.0)
Pneumonia^*^	21 (8.5)	12 (9.2)	9 (7.6)
Osteomyelitis	18 (7.3)	15 (11.5)	3 (2.5)
Endocarditis	13 (5.2)	9 (6.9)	4 (3.4)
Bacteremia^+^	41 (16.5)	33 (25.4)	8 (6.8)
Epidural abscess	1 (0.4)	1 (0.8)	0 (0)
Necrotizing fasciitis/myositis	3 (1.2)	3 (2.3)	0 (0)
Fungemia	4 (1.6)	2 (1.5)	2 (1.7)
** ≥** 1 infection	159 (64.1)	130 (100)	29 (24.6)
**Other Infection –** ***no.*** **(%)**	54 (21.8)	25 (19.2)	29 (24.6)
**Chronic Infection** ***– no. (%)***			
Hep C	82 (33.1)	45 (34.6)	37 (31.4)
HIV	24 (9.7)	14 (10.8)	10 (8.5)
**Received ID Consultation** ***– no. (%)***	82 (33.1)	57 (43.8)	25 (21.2)

Abbreviations: Hep, hepatitis; HIV, human immunodeficiency virus; ID, infectious disease; IDU, injection drug-use; OUD, opioid use disorder; SSTI, skin and soft tissue infection

^*^Pneumonia unrelated to OUD as described in the patient’s EMR, such as hospital-acquired pneumonia, is categorized as “other infection.”

^+^Bacteremia unrelated to OUD as described in the patient’s EMR, such as bacteremia secondary to colitis, is categorized as “other infection.”

**Table 3:** Distribution of microorganisms in patients with IDU-Associated infections

	All Patients (*N* = 130)
**Gram-positive cultures – no. (%)**	
Methicillin-Resistant *Staphylococcus aureus*	31 (23.8)
Methicillin-Susceptible *Staphylococcus aureus*	28 (21.5)
Coagulase-negative *Staphylococcus spp.*	7 (5.4)
* Streptococcus spp.*	39 (30)
* Streptococcus anginosus*	5 (3.8)
* Streptococcus constellatus*	9 (6.9)
* Streptococcus pyogenes*	9 (6.9)
* Clostridium spp.*	7 (5.4)
* Enterococcus spp.*	4 (3.1)
Other Gram-positives	18 (13.8)
**Gram-negative cultures** ***– no. (%)***	
* Bacteroides spp.*	3 (2.3)
* Enterobacter spp.*	2 (1.5)
* Haemophilus parainfluenzae*	2 (1.5)
* Klebsiella pneumoniae*	6 (4.6)
* Pseudomonas aeruginosa*	3 (2.3)
* Proteus spp.*	3 (2.3)
Other Gram-negatives	7 (5.4)
**Other** ***– no. (%)***	
* Candida spp.*	7 (5.4)
Other fungi	2 (1.5)
Nontuberculous *Mycobacterium spp.*	1 (0.8)
**Positive Cultures** ***– no. (%)***	82 (63.1)
Monomicrobial infection	52 (40.0)
Polymicrobial infections	30 (23.1)

Abbreviations: IDU, injection drug-use

While fewer patients with IDU-associated infections were receiving MOUD on admission (Table 4, 7.7% vs. 32.2%, *p* < 0.0001), new inpatient uptake of MOUD was higher in patients with IDU-associated infections (80.8% vs. 50.8%, *p* < 0.05). Follow-up attendance was lower in patients with IDU-associated infections (Table 5, 27.5% vs. 39.0%, *p* = 0.059).

**Table 4:** MOUD Uptake and referral

	All Patients (*N* = 248)	IDU-Associated Infection (*N* = 130)	No IDU-Associated Infection (*N* = 118)
**Active MOUD On Admission** ***– no. (%)***	48 (19.4)	10 (7.7)	38 (32.2)
**No Uptake** ***– no. (%)***	35 (14.1)	15 (11.5)	20 (16.9)
Patient Refused	24 (9.7)	11 (8.5)	13 (11.0)
Other*	11 (4.4)	3 (2.3)	7 (5.9)
**New MOUD Uptake** ***– no. (%)***	165 (66.5)	105 (80.8)	60 (50.8)
Methadone	54 (21.8)	38 (29.2)	16 (13.6)
Buprenorphine	97 (39.1)	62 (47.7)	35 (29.7)
Buprenorphine/Naloxone	9 (3.6)	4 (3.1)	5 (4.2)
Naltrexone	5 (2.0)	1 (0.8)	4 (3.4)
**Outpatient Referral** ***– no. (%)***			
None	56 (22.6)	28 (21.5)	28 (23.7)
Drug Rehab	21 (8.5)	14 (10.8)	7 (5.9)
Outpatient MOUD	152 (61.3)	78 (60.0)	74 (62.7)
Support Services	17 (6.9)	9 (6.9)	8 (6.8)
Unknown	2 (0.8)	1 (0.8)	1 (0.8)

Abbreviations: IDU, injection drug use; MOUD, medication for opioid use disorder

*Patient MOUD treatment was not initiated for a reason other than refusal, e.g. the patient was not deemed a good candidate for MOUD, patient-directed discharge before initiating MOUD

**Table 5:** Discharge disposition and follow up

	All Patients (*N* = 248)	IDU-Associated Infection (*N* = 130)	No IDU-Associated Infection (*N* = 118)
**Discharge Disposition** ***– no. (%)***			
Home/Shelter	162 (65.3)	74 (56.9)	88 (74.6)
Jail	15 (6.0)	9 (6.9)	6 (5.1)
Skilled Nursing Facility	18 (7.3)	12 (9.2)	6 (5.1)
Inpatient Facility	8 (3.2)	4 (3.1)	4 (3.4)
Patient-Directed Discharge	30 (12.1)	21 (16.2)	9 (7.6)
Other/Unknown	15 (6.0)	10 (7.7)	5 (4.2)
**Follow-Up Attendance** ***– no. (%)***			
No Attendance	103 (41.5)	57 (43.8)	46 (39.0)
Attendance	82 (33.1)	36 (27.5)	46 (39.0)
No Follow-Up Scheduled	60 (24.2)	35 (26.7)	25 (21.2)
Unknown	3 (1.2)	3 (2.3)	0 (0)
**ED Visit Within 90 Days**			
ED Visit Within 90 Days – *no. (%)*	107 (43.1)	59 (45.4)	48 (46.7)
Mean ED Visit Frequency – *no.*	2.1 ± 1.6	2.2 ± 1.9	2.0 ± 1.2
Readmission Within 90 Days – *no. (%)*	64 (25.8)	34 (26.2)	30 (25.4)

Abbreviations: ED, emergency department; IDU, injection drug-use

In univariate analysis, having a diagnosis of anxiety was associated with lower odds of starting MOUD (*p* < 0.05) in those with an IDU-associated infection compared to those without an infection. Additionally, the following were each associated with greater odds of starting MOUD in those with an IDU-associated infection: having a history of intravenous injection (*p* < 0.05), having an infection requiring intravenous antibiotics (*p* < 0.05), or receiving a referral to a peer navigator (*p* < 0.05). Multivariate analysis showed a negative association with anxiety (aOR = 0.372, 95% CI [0.144–0.958], *p* < 0.05) and a statistically insignificant positive association with IDU-associated infections (aOR = 2.126, 95% CI [0.961–4.706], *p* = 0.0627).

Skin popping (*p* < 0.05), infective endocarditis (*p* < 0.01), and outpatient intravenous antibiotics (*p* < 0.01) were positively associated with rehospitalization in univariate analysis. Multivariate analysis showed a negative association with cannabis use (aOR = 0.473, 95% CI [0.224–0.999], *p* < 0.05) and a positive association with discharge to a skilled nursing facility (aOR = 5.732, 95% CI [2.036–16.134], *p* < 0.001). IDU-associated infections (aOR = 0.909, 95% CI [0.484–1.706], *p* = 0.7655) and any MOUD uptake (aOR = 1.163, 95% CI [0.473–2.861], *p* = 0.7427) were not associated with rehospitalization.

## Discussion

Our study characterizing infections among hospitalized patients with OUD in a southern safety-net hospital identified SSTIs as the most common infection type in this population. SSTIs were 6 times more common among those with recent IDU (81.5% v 11.0%). In addition, a substantial proportion of infections were polymicrobial, including streptococcal, staphylococcal, Gram-negative, anaerobic, and fungal organisms. Taken together, these findings underscore the unique epidemiology that is seen among PWID with OUD in a region where black tar heroin has been more prominent.

Similar studies of infections among people with OUD in other parts of the country such as Florida identified a much higher proportion of infective endocarditis (9.5%) and bacteremia (52%) versus 6.9% and 25.4% in our study [27]. As these infections are more typically associated with intravenous heroin use compared to intramuscular subcutaneous use, these higher rates likely reflect higher rates of white powder heroin and intravenous heroin use in Florida [28, 29]. Studies in North Carolina and Oregon have indicated dramatic increases in infective endocarditis, central nervous system infections, and bacteremia [30, 31]. Beyond geographic distribution, route of administration plays an important role; a study in Boston demonstrated higher rates of past-year SSTIs in PWID who administer subcutaneously versus intravenously though this region has predominantly white powder heroin [32].

Few other studies have identified the variety of pathogens, including Gram-negatives and anaerobes, as we did in this study. Previous microbial profiling of SSTIs in both people with and without IDU demonstrate a predominance of *Staphylococcus aureus* (76–83%) and *Streptococcus* species (7%) with predominantly monomicrobial infections [33, 34]. Our findings are consistent with growing literature showing increased prevalence of polymicrobial and anaerobic SSTIs in PWID [35, 36]. This has important implications for empiric antimicrobial therapy in this setting, which would need to treat organisms beyond common gram-positives and may require surgical incision and drainage. In addition, healthcare providers in areas where black tar heroin is used need to remain vigilant about the possibility of life-threatening clostridial infections, including botulism, tetanus, and others [37–39].

Our study had several other unique epidemiologic findings. We had a lower proportion (48%) of non-Hispanic White patients in our cohort compared to 69% of people who inject heroin in Baltimore City and over 80% in people who use opioids in Florida [27, 40]. In addition, our study further demonstrates increasing rates of concurrent stimulant use: 77% of patients with IDU-associated infections also used stimulants, compared to 22% in a study of hospitalizations for IDU-associated infections in Florida. As opioids and stimulants may mask clinical presentations of each other, concurrent use is associated with increased rates of fatal overdose [41, 42]. Furthermore, concurrent use of stimulants is associated with worsened substance use treatment outcomes [43, 44].

In this study, those with IDU-associated infections were less likely to be taking MOUD before hospitalization than those without these infections (7.7% vs. 32.2%), which may indicate the key role MOUD plays in harm reduction. High rates of MOUD uptake, especially amongst those with IDU-associated infections, support the notion that hospitalization can be an effective circumstance in which to initiate substance use treatment. In fact, at the time of discharge 84.7% of all patients in this study were receiving MOUD. Intravenous route of administration of opioids and infection requiring intravenous antibiotics were associated with new MOUD uptake and anxiety was a negative predictor of MOUD uptake. Other studies have shown increased MOUD uptake among individuals experiencing homelessness and individuals who have a partner who uses substances, however methamphetamine use was negatively associated with MOUD uptake [45].

MOUD uptake has many important implications for harm reduction. All forms of MOUD (i.e. methadone, buprenorphine, naltrexone) are associated with a reduction in injection frequency, as well as improved outcomes for people living with HIV and hepatitis C, including improved adherence to antiretrovirals, adherence to directly acting antivirals, HIV virologic suppression and completion of hepatitis C treatment [46–53]. In addition, MOUD initiation or continuation during acute infection may facilitate care engagement among patients admitted with IDU-associated infections, including integration of infectious diseases and addiction care as well as increased eligibility for self-administered OPAT and participation in other complex aspects of their own medical care [54, 55]. These interventions can result in shortening patient stays, decreasing costs, and improving patient satisfaction [56].

Our study is not without limitations. Ours is a single site descriptive study conducted at a large urban hospital in the southern US and therefore our findings may not apply to dissimilar settings. However, we describe a critical gap in the literature as few other studies of infections among PWID have focused on this geographic area, with implications for other states where black tar heroin use is historically prevalent such as California and Washington. Second, the identification of microorganisms associated with SSTIs was limited by the paucity of conclusive cultures in this infection and therefore likely underestimates the diversity of causative organisms. Additionally, our data is subject to the selection bias of only including those with recognized OUD who received addiction psychiatry consults, which could exclude those with undiagnosed or unrecognized OUD who did not receive consults and may be underrepresented by ICD codes. Lastly, we had incomplete follow-up data on whether patients continued MOUD following discharge, as our study was limited to our institutional EMR. Future studies should examine retention in MOUD and continuity of clinical services after hospitalization in this population as this is a critical transition of care [57, 58].

## Conclusions

SSTIs were the most prevalent IDU-associated infection in hospitalized patients with OUD in an urban, safety-net hospital in the South, with bacteremia, osteomyelitis and endocarditis representing a much smaller proportion of infections. This finding is most likely related to high rates of black tar heroin use, which is associated with skin-popping and intramuscular administration. Furthermore, infectious microbes causing these infections included gram-negative, anaerobic, and fungal organisms, indicating a need for broad empiric antibiotic treatment and early surgical evaluation. High rates of polysubstance use with both opioids and stimulants in this population require monitoring and education around possible overdose. Our findings also highlight the need for increased access to harm reduction services in the South. Importantly, hospitalization for IDU-associated infections among people with OUD represents a critical time for MOUD initiation and continuation, though further studies are needed to examine continuity of MOUD after discharge and longer-term clinical outcomes.

## Data Availability

The datasets analyzed during the current study are available from the corresponding author on reasonable request.

## Abbreviations

HIV: Human immunodeficiency virus
IDU: Injection drug use
MOUD: Medication for opioid use disorder
OPAT: Outpatient parenteral antimicrobial therapy
OUD: Opioid use disorder
PTSD: Post-traumatic stress disorder
PWID: People who inject drugs
SSTI: Skin and soft-tissue infection
